# Management of Chronic Pain by Occupational Therapist: A Description of Practice Profile

**DOI:** 10.1177/00084174231162709

**Published:** 2023-03-20

**Authors:** Émilie Lagueux, Julie Masse, Raphaël Pagé, Béatrice Marin, Yannick Tousignant-Laflamme

**Keywords:** Assessments, Chronic pain, Health care survey, Interventions, Practice process, Sondage en ligne, douleur chronique, processus de pratique, évaluations, interventions

## Abstract

**Background.** Current state of knowledge regarding occupational therapy's contribution to chronic pain (CP) management has evolved over the past decade. Yet, has this been transferred to clinical practice? **Purpose.** Describe the current state of practice of CP management-specific occupational therapy. **Method.** An online survey was sent to occupational therapists working with CP patients. **Findings.** Of the 90 respondents (11.9%), 42.2% worked in primary care and 52.2% in secondary care. They reported that their primary role aimed at enabling occupation and providing vocational rehabilitation. The Canadian Model of Occupational Performance and Engagement (CMOP-E) (87.8%), semi-structured interview (86.7%), and education on energy conservation (65.6%) and postural hygiene (60.0%) were the most frequently reported conceptual model, assessment, and intervention methods. **Implications.** Results illustrate the diversity of current occupational therapy practice in CP management and suggest opportunities for improvement to ensure best practices are adopted, by emphasizing an occupation-based vision of health and well-being.

## Résumé

**Description**. L'état actuel des connaissances concernant la contribution de l'ergothérapie en gestion de la douleur chronique (DC) a évolué dans la dernière décennie. Cela a-t-il été transféré à la pratique clinique. **But.** Décrire l'état actuel de la pratique ergothérapique en gestion de la DC. **Méthodologie.** Un sondage en ligne a été envoyé aux ergothérapeutes. **Résultats.** Sur les 90 répondants (11,9%), 42,2% travaillaient principalement en soins primaires et 52,2% en soins secondaires. Leur rôle principal visait à promouvoir l'occupation et la réadaptation au travail. Le Modèle canadien du rendement et de l'engagement occupationnel (87,8%), l'entrevue semi-structurée (86,7%) et l'éducation sur la conservation d'énergie (65,6%) et l'hygiène posturale (60,0%) étaient les plus fréquemment cités. **Conséquences.** Les résultats illustrent la diversité de la pratique ergothérapique actuelle et suggèrent des possibilités d'amélioration, en mettant l'accent sur une vision de la santé et du bien-être fondée sur l'occupation.

## Introduction

Chronic pain (CP) is defined by the International Association for the Study of Pain (IASP) as pain that persists or recurs for longer than 3 months and represents a major public health burden (Treede et al., 2015). For long considered as a symptom, the World Health Organization has officially recognized CP as a diagnosis in its own right in the 11th revision of the International Classification of Diseases (ICD-11) (Treede et al., 2019). In Canada, large population-based surveys have estimated that one in five Canadians will live with CP at some point in his/her lifetime (Campbell et al., 2021).

It has become essential to adopt biopsychosocial models of pain to enhance the understanding of CP and acknowledge each individual's experience of pain and its impact on their lives (Campbell et al., 2019, 2021; Raja et al., 2020). In fact, many individuals living with CP present limitations in their ability to perform meaningful activities of daily living that negatively affect their quality of life and well-being (Campbell et al., 2021; Dueñas et al., 2016). These persons need effective and individualized pain management. However, providing such care is complex and involves a wide range of treatment modalities and approaches that often exceed the expertise of a single profession (Campbell et al., 2019). Thus, multidisciplinary treatments including physical, psychological, and pharmacological therapies are now recognized as the gold standard for effective pain management, and specialized pain clinics are an important element within its spectrum (Campbell et al., 2019).

As members of specialized CP management clinics (IASP, 2018), occupational therapists provide distinct value in the management of CP by directly targeting the person's engagement in significant occupations and using occupation itself as a medium for therapy (Hill, 2016; Lagueux et al., 2018). Occupational therapy is strongly anchored in the biopsychosocial model of health and supports active engagement in meaningful occupations for individuals living with CP (American Occupational Therapy Association [AOTA], 2021; Strong, 1996). Indeed, persons living with CP believe that lifestyle is an important factor in health-related quality of life and 92% of those entering a multidisciplinary pain clinic report being motivated to change their lifestyle (Nielsen et al., 2021). Thus, individually tailored multimodal interventions should include occupational approaches addressing relevant lifestyle factors associated with CP (Nijs et al., 2020). Knowledge about occupational therapy's unique contribution to CP management is evolving. Recent scoping reviews (Lagueux et al., 2018) and guidelines (AOTA, 2021) shed some light on the current state of knowledge regarding occupational therapy's roles, models, assessments, and intervention methods in CP management. However, little is known about the clinicians’ current practice. The purpose of this study is to describe the current state of practice of occupational therapists working with persons suffering from CP in the Canadian health care system (province of Quebec).

## Method

### Design

A cross-sectional observational study was conducted, where descriptive information was gathered via an online survey.

### Participants

For this descriptive study, occupational therapists working in CP management were recruited. To be included in the study, potential participants had to (a) hold a valid occupational therapy license from the provincial registrar the Ordre des Ergothérapeutes du Québec (OEQ) (provincial licensing organization) and (b) be working (providing care) in a context of CP management with an adult population.

### Recruitment Procedures

While renewing their annual license, Quebec's occupational therapists are asked to state their practice profile and the population they serve (i.e., CP management with an adult population). This specific subgroup (occupational therapists meeting the inclusion criteria, *n* = 755) was targeted and email invitations to participate in this survey were sent via the OEQ platform to potential eligible participants. The email included a brief description of the project, the hyperlink to the survey, instructions to complete the questionnaire, as well as information to contact the main investigator, if necessary. Simultaneously, the invitation message and link to the online survey were posted on the Ergothérapie QUÉBEC Facebook page. Three weeks later, a reminder was sent by email by the OEQ and via the Facebook page. As an incentive, 10 gift certificates were randomly attributed to participants. The survey was closed 5 weeks after being posted. All information was in French, and data were collected from February 2019 to March 2019.

### Ethical Considerations

All participants provided consent through the online survey platform and their data (responses) were kept anonymous using an alphanumeric codification.

### Data Collection

Data were obtained through an online survey platform (LimeSurvey™). The structure and content of the survey were designed by a research team comprised of two research experts in CP management and six occupational therapy graduate students. It was built using the custom design method (de Leeuw et al., 2008), the available literature on the role of occupational therapy in CP management and the IASP Curriculum Outline on Pain for occupational therapy (IASP, 2018). The survey was formally pilot tested with occupational therapists deemed experts in CP management from three different settings. To reflect the clinical reality of occupational therapists, the survey was divided into two sections following the Canadian Practice Process Framework (Townsend & Polatajko, 2014). Initial screening questions enabled the application of the inclusion criteria. The first section of fthe survey addressed the practice context and had nine (15) close-ended questions that captured the sociodemographic characteristics of the professionals and characteristics of both their clientele, their practice environment, the main source of referral client, duration of follow-up, discharge criteria, and health care professionals with whom they collaborate most frequently. The second section of the survey addressed the practice process and had seven (10) close-ended questions that identified the principal role, use of practice models, evaluation, and five most frequently used intervention of participants regarding their practice process and need competency development. Occupational therapists’ perception of their own level of knowledge and competency regarding the various aspects of their practice in CP management was assessed through an 11-point numeric scale (0 = very weak and 10 = very strong).

### Data Analysis

Descriptive statistics (mean and *SD* for continuous variables; frequency and percentages for categorical variables) were used to analyse and report the quantitative data collected by the close-ended questions using Excel. Participants’ scores regarding the perception of their own level of knowledge and competency were arbitrarily converted into three subcategories where scores <5 were considered as “weak”, scores between 5 and <8 were considered as “moderate”, and scores from 8 to 10 were considered as “high” perceived competency (Aithal et al., 2019).

## Results

### Participants’ Demographics

Among the 755 occupational therapists solicited by the OEQ, a total of 90 participants (11.9%) completed all the survey questions. Occupational therapists who completed the survey had an average of 12.8 ± 10.0 years of experience in occupational therapy, 8.9 ± 7.5 years of experience working in CP management, and were mainly women (*n* = 84, 93.3%). Half of the participants (*n* = 45, 50.0%) worked in the Montreal Metropolitan Area. Table 1 provides additional information about participants’ demographics.

**Table 1:** Demographics of Occupational Therapists

Demographic attributes	Respondents (*n* = 90)	
	*n* (%)	
Gender		
Women	84	(93.3)
Men	5	(5.6)
Prefer not to respond	1	(1.1)
Entry-level diploma		
Bachelors	46	(51.1)
Masters	43	(47.8)
Specialized graduate diploma	1	(1.1)
Diploma obtained from		
Université de Montréal	39	(43.3)
Université Laval	23	(25.6)
McGill University	9	(10.0)
Université de Sherbrooke	8	(8.9)
Université d’Ottawa	5	(5.6)
Université du Québec à Trois-Rivières	4	(4.4)
McMaster University	1	(1.1)
Unspecified	1	(1.1)
Administrative regions of their work setting		
Montréal metropolitan area	30	(33.3)
Montérégie	15	(16.7)
Capitale Nationale	8	(8.9)
Estrie	7	(7.8)
Laurentides	6	(6.7)
Chaudière-Appalaches	5	(5.6)
Gaspésie-Iles-de-la-Madelaine	3	(3.3)
Mauricie	3	(3.3)
Outaouais	3	(3.3)
Saguenay-Lac-Saint-Jean	3	(3.3)
Abitibi-Témiscamingue	2	(2.2)
Centre-du-Québec	2	(2.2)
Lanaudière	2	(2.2)
Côte-Nord	1	(1.1)
Years of experience (yrs ± *SD*; [range])		
As an OT	12.8 ± 10.0; [1–41]	
As an OT working in CP	8.9 ± 7.5; [1–35]	

Abbreviations: CP = chronic pain; OT = occupational therapists; *SD* = standard deviation; Yrs = years.

### Practice Context

**Figure 1.**
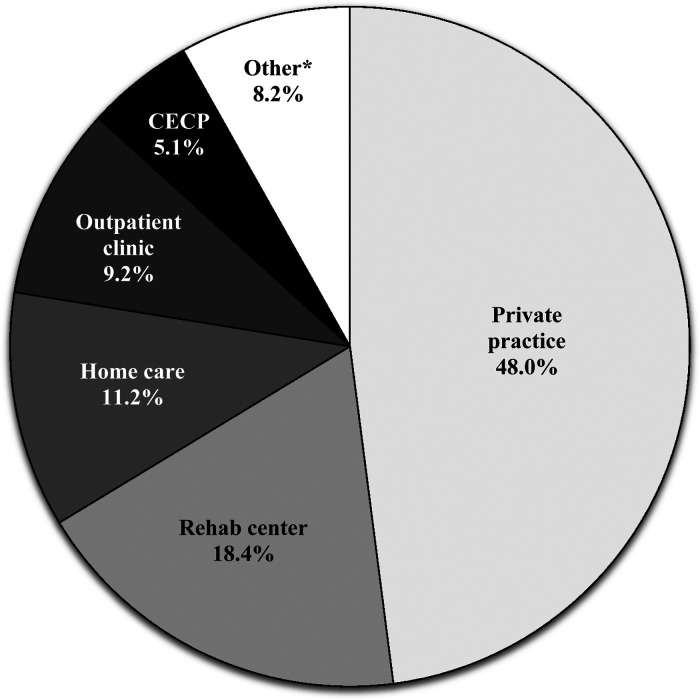
Work settings reported by the occupational therapists surveyed. *Other includes : Hospital Center (care unit); Residential and Long-Term Care Centers (CHSLDs); Intensive Functional Rehabilitation Unit (URFIs); Day Hospital; Canada Veterans advisory team; Human Resources, Communications and Legal Affairs Directorate (HRDCAJ) for health network employees' disabilities prevention; military health center. Abbreviation: CECP = Centre of Expertise in Chronic Pain.

Based on the Quebec organization of health care services, participants reported that they mainly worked in primary care (*n *= 38, 42.2%) or in secondary care (*n *= 47, 52.2%), whereas tertiary care was reported by 22.2% (*n *= 20) of respondents. Private practice was the most frequently reported work setting (Figure 1). Occupational therapists reported collaborating with a variety of health care professionals within a multidisciplinary (*n *= 59, 65.6%) or interdisciplinary (*n* = 62, 68.9%) rehabilitation team (Table 1). Independently of their work settings, participants were asked to specify the environment in which they provide care: 81.1% (*n* = 73) of the participants indicated to frequently or always provide care in a clinical setting, 25.6% (*n *= 23) reported to frequently or always provide care at their client's home and 10% (*n* = 9) reported to frequently or always provide care at their clients’ workplace.

Results suggest that most occupational therapists intervene 1–3 times per week on an individual basis (*n = *57, 63.4%). Regarding the use of group interventions, 65.6% of the participants (*n *= 59) indicated that it was not applicable in their context of practice. Among those who mentioned integrating group interventions in their practice (*n* = 31, 34.4%), more than half (*n* = 16) reported doing so 1–3 times a week. Average duration of the intervention follow-up was 3 to 12 months for 57.8% (*n *= 52) and less than 3 months for 36.7% (*n *= 33) of respondents. Main sources of client referral and discharge criteria are shown in Table 2; achievement of objectives and lack of progress being the most reported discharge criteria.

**Table 2:** Description of the Practice Context

**Elements of the practice context**		**Number of respondents**
		**n^a^ (%)**
Main source of patient referral		
From a physician		67 (74,4)
Third-party payer (including CNESST and SAAQ)		54 (60.0)
Self-referred client		25 (27.8)
Another health care professional		25 (41.7)
Health institutions		12 (13.3)
Veterans’ affairs		4 (4.4)
Employer		1 (1.1)
Main criteria for discharge		
Achievement of client's objectives		81 (90.0)
Lack of progress		60 (66.7)
End of the service agreement		31 (34.4)
Client's needs falling within the competencies of another health care professional		29 (32.2)
Disengagement of the client in the intervention process		28 (31.1)
End of a program with a predetermined duration		27 (30.0)
Client's absence of needs		23 (25.6)
Client's request to end the follow-up		15 (16.7)
Lack of time or resources		7 (7.78)
Health care professionals with which survey respondents collaborate most frequently	Within a multidisciplinary team^b^	Within an interdisciplinary team^c^
	n = 59 (%)	n = 62 (%)
Physiotherapist	45 (76.7)	53 (85.7)
Technician in Physical Rehabilitation	23 (42.2)	24 (25.6)
Physician	38 (52.5)	23 (24.2)
Psychologist	29 (49.2)	26 (41.9)
Kinesiologist	22 (37.3)	32 (51.6)
Social worker	20 (33.9)	18 (29.0)
Nurse	21 (35.6)	12 (19.4)
Other OTs	18 (30.5)	22 (35.5)
Others*	8 (8.9)	8 (8.9)

**^a^** Sample sizes may vary as responders provided multiple answers to the same questions or not all responders provided a response to all questions.

^b^ Multidisciplinary team consists of a grouping of several stakeholders where each professional performs his or her task independently.

^c^ Interdisciplinarity team consists of a grouping of several stakeholders working in the synthesis where a harmonization between points of view is integrated into a coherent and coordinated whole.

*Others include psychotherapist, psychoeducator, specialized educator, recreational technician, nutritionist, guidance counsellor, and rehabilitation counsellor.

Abbreviations: CNESST = Committee on Standard, Equity, Health and Safety at Work; OTs = occupational therapists; SAAQ = Quebec Automobile Insurance Board.

**Figure 2.**
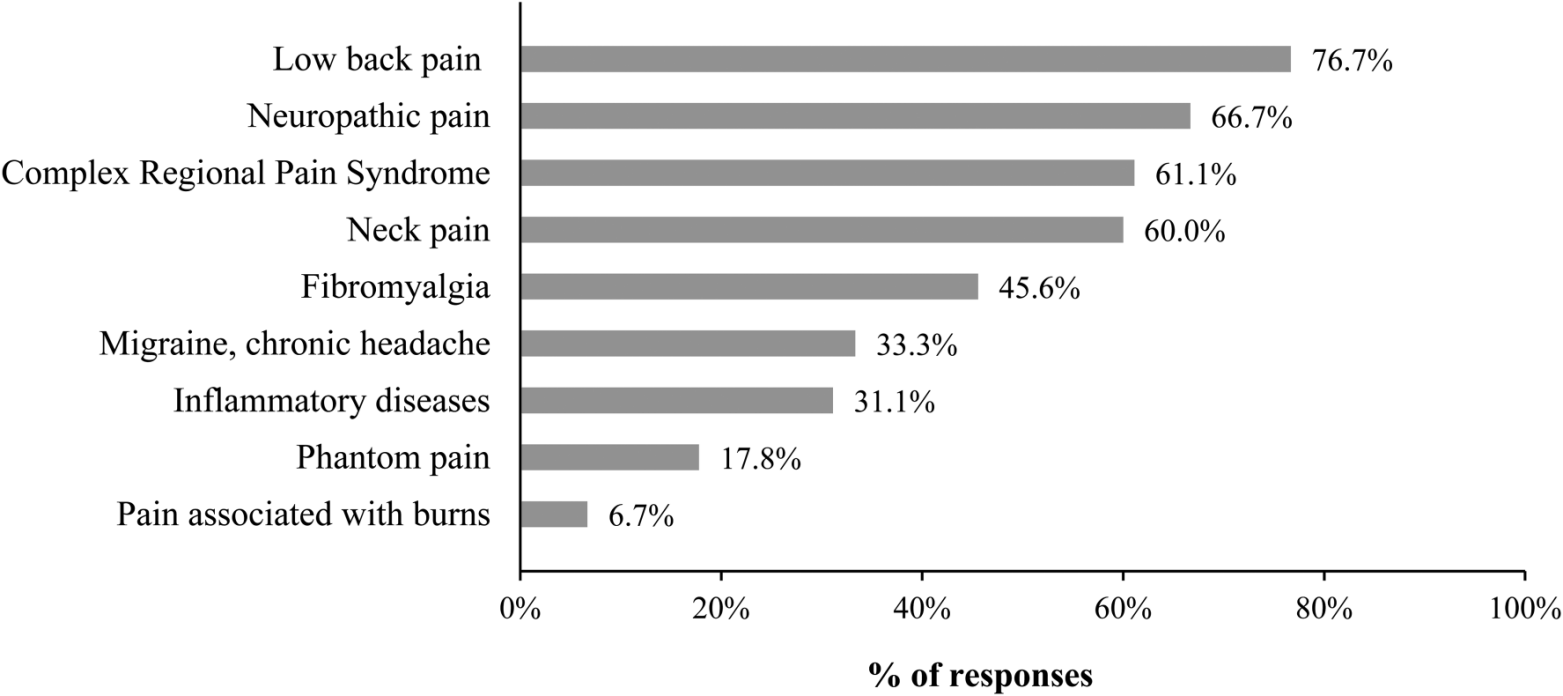
Clinical conditions (health problems) encountered by the occupational therapists surveyed.

Almost one in two respondents (*n *= 42, 46.7%) indicated that their caseload was comprised of more than 50% of clients living with CP. Occupational therapists reported that they encountered a wide variety of pain-related conditions in their CP management practice, such as low back pain (*n* = 69, 76.7%), neuropathic pain (*n* = 60, 66.7%), complex regional pain syndrome (*n* = 55, 61.1%), and neck pain (*n* = 54, 60.0%) (Figure 2). They reported a moderate perceived level of knowledge regarding their client's clinical conditions (6.95 ± 1.39/10) and the neurophysiology of pain (6.9 ± 1.7/10).

### Practice Process

Detailed results regarding the roles, conceptual models, assessments, and intervention methods supporting the practice process of occupational therapists in CP management, are shown in Table 3, and the main findings are summarized below**.**

**Table 3:** Description of the Practice Process

		Number of respondents
		n^a^ (%)
Roles	Enabling occupation	81 (90.0)
	Providing vocational rehabilitation	85 (94.4)
	Offering therapeutic education	78 (86.7)
	Grading and adapting activities	76 (84.4)
	Modifying environment and ergonomics	71 (78.9)
	Enhancing psychological support	67 (74.4)
Conceptual models used	CMOP-E	79 (87.8)
	PEO	38 (42.2)
	HDM-DCP	22 (24.4)
	MOHO	17 (18.9)
	None or unknown	16 (17.8)
	CIF	4 (4.4)
	Humanistic model	1 (1.1)
Assessment methods	Interview	
	Semi-structured interview	78 (86.7)
	Non-structured interview	25 (27.8)
	Structured interview	6 (6.7)
	Subjective pain evaluation	74 (82.2)
	Structured observation of occupational performance	
	In a simulated context	72 (80.0)
	In the client's real environment	64 (71.1)
	Activity analysis	65 (72.2)
	Standardized self-administered questionnaire	63 (70.0)
	Environment assessment	49 (54.4)
Intervention approaches	Interventions focusing on the person	
	Energy conservation education	59 (65.6)
	Postural hygiene education	54 (60.0)
	Stress management	31 (34.4)
	Exercise program	24 (26.7)
	Mindfulness	21 (23.3)
	Sensory rehabilitation	18 (20.0)
	Coping strategies education	12 (13.3)
	Mobilization techniques	8 (8.9)
	Therapeutic activities	7 (7.8)
	Mental imagery or visualization techniques	7 (7.8)
	Thermal modalities	6 (6.7)
	Massage	3 (3.3)
	Edema management	2 (2.2)
	Stress loading	1 (1.1)
	Acceptance and commitment therapy	1 (1.1)
	Education on symptoms	1 (1.1)
	Interventions focusing on occupation	
	Task adaptation	42 (46.7)
	Occupational schedule modification	35 (38.9)
	Activity pacing	33 (36.7)
	Task training	23 (25.6)
	Sleep routine	21 (23.3)
	Use of ergonomic or adapted equipment	13 (14.4)
	Interventions focusing on environment	
	Modification of the physical environment	20 (20.2)

^a^ Sample sizes vary as not all participants provided a response to all questions.

Abbreviations: ADL = activity of daily living; CMOP-E = Canadian Model of Occupational Performance and Engagement; HDM-DCP = The Human Development Model – Disability Creation Process; ICF = International Classification of Functioning, Disability and Health; IDAL = Instrumental activity of daily living; MOHO = Model of Human Occupation; PEO = Person-Environment-Occupation model; *SD* = standard deviation.

*Roles*: According to the participants, the main roles of occupational therapists in CP management are (1) enabling occupation (*n* = 85, 94.4%); (2) providing vocational rehabilitation (*n* = 81, 90%); (3) offering therapeutic education (*n* = 78, 86.7%); (4) grading and adapting activities (*n* = 76, 84.4%); (5) modifying environment and ergonomics (*n* = 71, 78.9%); and (6) enhancing psychosocial support (*n* = 67, 74.4%). Occupational therapists reported a moderate competency level regarding their role (6.7 ± 1.6/10).

*Conceptual models*: The Canadian Model of Occupational Performance and Engagement (CMOP-E) (*n* = 79; 87.8%) and the Person-Environment-Occupational (PEO) model (*n* = 38; 42.2%) have been reported to be the most widely used theoretical models among the participants in this survey. Occupational therapists reported a moderate competency level regarding the use of conceptual models (6.9 ± 2.0 /10).

*Assessment methods*: Occupational therapists reported a combination of numerous methods related to the assessment of their clients in CP: (1) semi-structured interview (*n* = 78, 86.7%); (2) subjective pain evaluation (*n* = 74, 82.2%); (3) occupational performance observation in a simulated context (*n* = 72, 80.0%); (4); occupational performance structured observation in the client's natural environment (*n* = 64, 71.1%); and (5) activity analysis (*n* = 65, 72.2%). Moreover, they reported a moderate competency level regarding the use of assessment methods (7.6 ± 1.4/10).

*Intervention approaches*: Clinical interventions used by occupational therapists were classified based on PEO (Table 3). The intervention methods that were reported by participants as being the most frequently used with their clients in CP management are (1) education on energy conservation (*n* = 59, 65.6%); (2) postural hygiene education (*n* = 54, 60.0%); (3) task adaptation (*n =* 42, 46.7%); (4) occupational schedule modification (*n *= 35, 38.9%); and (5) activity pacing (*n =* 33, 36.7%). They reported a moderate competency level regarding the use of intervention modalities (7.6 ± 1.4/10).

*Competency development*: As the main perceived needs concerning the development of competency, occupational therapists report a need course concerning: (1) the methods of intervention (*n *= 33, 36.7%); (2) the use of evidence-based clinical practice data (*n* = 22, 24.4%); (3) the specific role of occupational therapy in CP management (*n* = 13, 14.4%); (4) the conceptual models (*n* = 10, 11.1%); (5) the assessment methods (*n* = 8, 8.9%); and (6) the knowledge about the neurophysiology of pain (*n *= 4; 4.4%).

## Discussion

This is the first study to describe the current state of practice of occupational therapy in CP management in the province of Quebec (Canada). This survey reveals similarities with current scientific evidence and highlights areas for improving the competencies of occupational therapists in connection with recent guidelines (AOTA, 2021; IASP, 2018).

### Current Occupational Therapy Practice Contexts

This survey reveals that occupational therapists intervene with person affected by a variety of pain-related conditions and collaborate with numerous health care professionals, which is well aligned with recent clinical guidelines (AOTA, 2021; Campbell et al., 2019; IASP, 2018). Based on a biopsychosocial understanding of CP, treatment options should include physical, educational, psychological, social, and occupational components. According to Staudt (2022), there is no specific requirement for the composition of a multidisciplinary team, although the core players tend to include primary care providers such as physicians, psychologists, nurses, as well as physical and occupational therapists, which corroborates with what we observed in our results. It would be interesting to explore the nature and challenges of collaborative work between occupational therapists and other health care professionals such as physiotherapists and psychologists in future studies.

The Quebec (Canada) health care service system is based on a principle of service hierarchy, which facilitates the complementarity of services and the flow of person between first-, second-, and third-line services. Sharing a common philosophical basis with the profession of occupational therapy, primary health care aims to offer a variety of services for the treatment and prevention of disability, support a healthy lifestyle, promote health and increase social participation according to person's needs and preferences (as individuals, families, and communities) (Donnelly et al., 2023; World Health Organization, 2018).

Primary care is a relatively new practice setting for occupational therapists and offers a unique opportunity for the profession to draw on a health promotion perspective, as it has recently been demonstrated in a scoping review (Donnelly et al., 2023). Our results reported slightly less than half (42.2%) of occupational therapists working in primary care, mostly in private practice. None of the surveyed occupational therapists were involved in Family Medicine Groups, although this has been identified as a strategic clinical setting for optimal CP management pain (Campbell et al., 2019). Moreover, the review by Donnelly et al. demonstrated a breadth of contributions that occupational therapists are making in primary care, while focussing on understanding how individuals are engaging in everyday activities and supporting them to participate in their daily activities by building or adapting individual capacity, engaging in occupations, and ensuring safe and accessible environments (Donnelly et al., 2023). Even though physicians recognize several benefits of integrating occupational therapists into Family Medicine Groups (e.g., facilitate return to work, carry out group interventions on activation, healthy lifestyles, and pain management) (Locas et al., 2020), occupational therapists are still under-integrated into primary care teams (Dahl-Popolizio & Rogers, 2017); Donnelly et al., 2023; Locas et al., 2020). In Quebec, this might be explained by a lack of visibility and knowledge about the role of occupational therapists can play in CP management teams (Locas et al., 2020). According to Quebec physicians, the inclusion of occupational therapists in family medicine groups could help primary care teams address many of their clients’ needs and improve the overall quality and relevance of primary care services. Targeted strategies are thus needed to promote the integration of occupational therapists into this practice context (Locas et al., 2020).

Approximately half of the responders (52.2%) worked in Quebec secondary care settings. A large majority of them reported vocational rehabilitation being one of their main roles. These results corroborate with well-established occupational therapy services and research for many years which place a strong focus on return to work in secondary care settings with various populations (Désiron et al., 2011). Evidence highlights the fact that CP not only negatively affects work, but all occupational areas of a person's life (AOTA, 2021; Campbell et al., 2019; Hill & Macartney, 2019; Reyes & Brown, 2016). Moreover, results from a recent scoping review (Lagueux et al., 2018) revealed that occupational therapists’ roles go beyond providing vocational rehabilitation, since it includes enabling occupational engagement and performance at large, addressing occupational balance, restoring occupational identity, and limiting occupational injustice, despite pain.

Access to occupational therapy is another challenging issue, as many individuals must wait for several months, even years, to benefit from interdisciplinary specialized care (Campbell et al., 2021). Moreover, it has been found that occupational therapists are rarely involved in tertiary care settings in Quebec (Veillette et al., 2005). Even 10 years later, this finding still corroborates with our results, which reveal that only 22.2% of surveyed occupational therapists intervene in third-line clinical settings. Reasons for actual underrepresentation of occupational therapists in CP interdisciplinary care is unclear, but worth exploring. We can expect that issues like those that have been documented in the primary care context might be present in the third-line clinical context.

Although achievement of clinical objectives is by far the main reason for discharge, lack of progress, disengagement of the client in the intervention process, as well as pragmatic reasons, were also mentioned. These findings suggest that criteria used to measure clinical progress and factors influencing clients’ compliance with the proposed intervention need to be explored. If the focus is being placed first and foremost on symptoms, both clients and clinicians might be disappointed when pain intensity doesn’t significantly decrease in a short period of time. Helping clients make a cognitive shift from pain relief to pain management is challenging, but also empowering. Recent findings show that occupation-based interventions, which directly target the establishment of healthy routines (lifestyle changes), showed a trend for pain to remain stable, while participants’ daily functioning in meaningful activities improved (Lagueux et al., 2021; Simon & Collins, 2017). Therefore, according to these lifestyle-oriented interventions, instead of focusing exclusively on pain and disabilities, occupational therapy’s entire clinical process should also document and address links between meaningful occupations and well-being despite pain. This seems to be an interesting future avenue to explore.

### Main Features of Occupational Therapy Clinical Process

Describing the key features of occupational therapists’ clinical process is crucial given the diversity of clinical settings in which they can contribute to optimal CP management.

*Role*: In 2011, Robinson et al. 2011 warned that contemporary occupational therapy did not sufficiently put forward its specificity in CP management. However, occupational therapists surveyed in the present study clearly state being experts in enabling occupation with person living with CP, which is directly in line with the foundations of their profession (AOTA, 2021; Lagueux et al., 2018). In the past decade, the updating of clinical guidelines about occupational therapists’ role in improving occupational engagement (AOTA, 2021; IAPS, 2018) and evidence about field-specific interventions (Lagueux et al., 2018, 2021; Nielsen et al., 2021; Simon & Collins, 2017) improved understanding and promoted their distinct role in CP management. Hence, this survey reveals that clinicians are fully aware of their specificity and need to be supported to better deploy it throughout their clinical process. Promoting better recognition of the profession’ s added value, its benefits for persons with CP and opportunities to deploy its full potential is a topical issue (Turner & Knight, 2015; Walder et al., 2022).

*Conceptual model*: Consistent with the biopsychosocial model, “occupational therapy practitioners recognize the importance and impact of the mind–body–spirit connection on engagement and participation in daily life” (AOTA, 2020, pp. 6–7). In that sense, occupational therapy's role in pain management certainly is supported by a clear compatibility between occupational therapy's foundations and consensual recommendations for efficient CP management (AOTA, 2021). Conceptual models specific to the field of occupational therapy go even further than the biopsychosocial model by allowing a holistic understanding of the occupational challenges that individuals with CP face daily (Wong & Fisher, 2015). The CMOP/CMOP-E (Polatajko et al., 2007) is by far most frequently used by surveyed occupational therapists and was also mostly mentioned in a recent scoping review (Lagueux et al., 2018). Nevertheless, other disciplinary models deserve our attention since they provide a deeper understanding of some aspects of living with CP and the process of occupational adjustment. For example, the Model of Human Occupation (MOHO) (Taylor & Kielhofner, 2017) which is already used by some surveyed clinicians, guides occupational therapy practice by focusing on how individuals living with CP manage to build a positive occupational identity despite pain (Johansson et al., 2018). Such disciplinary conceptual models provide a rigorous understanding of the complexity of living with CP.

*Assessment methods*: Occupational therapists’ assessment process specifically allows the understanding of the “interaction between multiple personal, environmental, and occupational factors that explain the gap between what a person living with CP wants and needs to do, and their level of participation” (Lagueux et al., 2018, p. 16). To achieve this complex assessment, occupational therapists surveyed in the present study combine multiple assessment methods (interview, observation, questionnaires). The use of standardized tools specific to the field of occupational therapy, with an emphasis being placed on occupational participation in real-life situations, remains to be explored, since this study was unable to capture this information. Validated tools, such as the Canadian Occupational Performance Measure (COPM) (Law et al., 2014) and the Occupational Performance History Interview-II (OPHI-II) (Kielhofner, 2004), are essential to triangulate data collected by the other assessment methods and document clinical progress.

*Intervention approaches*: As listed in the scoping review (Lagueux et al., 2018), most of the intervention methods identified in this survey focus on the person's physical, cognitive, and affective dimensions. Many clinicians also reported significant results addressing occupation adjustments through task adaptation, schedule modification, and activity pacing, which is aligned with occupational therapy's distinct value in the treatment of pain using occupation itself as a medium for therapy (AOTA, 2021; Hill, 2016; Lagueux et al., 2018; Nielsen et al., 2021). In CP management, occupation, including activities of daily living and instrumental activities of daily living, may be used in training the client in concepts such as pacing; energy conservation; health management routines, such as exercise and sleep; body mechanics; and posture. Occupational therapy facilitates the integration of individualized self-management strategies into client's daily routine (AOTA, 2021). Whether to prevent the onset of chronicity, or to optimize treatment compliance and benefits, putting an emphasis on participation and lifestyle changes is complementary to interventions that seek the reduction of symptoms and disabilities more directly (Lagueux et al., 2021; Nielsen et al., 2021; Simon & Collins, 2017).

Modification of the physical environment has also been reported by surveyed clinicians. To ensure effective and sustainable day-to-day pain management strategies, the individual's social, cultural, and institutional environments should also be taken into consideration. Recent studies illustrate how social support represents an important issue to be addressed in CP management (Chou et al., 2018; Cooper & Gilbert, 2017) and should not be neglected when looking to improve participation in valued occupations. It has also become known that the sense of belonging to a group is associated with improved mental health for individuals living with CP (Lagueux et al., 2021; Sturgeon et al., 2015). However, our results suggest that occupational therapists rarely consider the social environment in their interventions, and seem reluctant to use group interventions, although these are highlighted in the recommendations of the guidelines of the IASP (IASP, 2018). Although the U.S. Department of Health and Human Services (2019) noted that support groups can increase access to care for persons with pain, it appears that the use of group interventions was not applicable for many participants, among other things, due to the context of practice. It could be partly due to organizational constraints, or to a need for improved animation skills of the occupational therapist. Nevertheless, a recent systematic review suggested that further high-quality and well-designed research is required to determine which elements of occupational therapy interventions most effectively influence the occupational engagement of individuals with CP (Griffiths et al., 2021).

*Competency development*: Surveyed occupational therapists reported a moderate competency level regarding their entire clinical process. More specifically, they expressed that professional development needs to rely on sound scientific evidence to articulate and justify their interventions. Indeed, suboptimal access and use of clinical research evidence have been documented in occupational therapy, as well as for various disciplines involved in CP management (Arumugam et al., 2018). Clinicians’ needs obviously reflect research gaps that should be filled in the near future.

### Strengths and Limitations of the Study

We are confident that this survey allows an appreciation of the current practice in CP management of occupational therapists in Quebec. In addition to exploring occupational therapists’ clinical process, it also considers their context of practice. Since the survey was formally pilot tested, we can assume that the questions were clear and relevant to participants. Nevertheless, some limitations must be acknowledged. Considering our low participation rate (12%), the study's sample size was relatively small. The largest group of respondents was observed in one metropolitan area. Thereby, it is not possible to confirm that the results in this study are representative of the actual practice of occupational therapists in CP management across Quebec. Moreover, our survey did not ask about the nature of the reference for occupational therapy services, which would have made it possible to obtain a more complete description of current practice. Finally, we must acknowledge that we considered occupational therapists’ perception of their own level of knowledge and competency regarding the various aspects of their practice in CP management using a continuous 11-point numeric scale and arbitrarily converted these continuous data into categorical data. Those results suggest clues about perceived needs in terms of continuing training more than they reflect the exact level of competency since self-report measures are not enough.

## Conclusion

This study describes the current practice of occupational therapy in CP management in Quebec (Canada). Results show that occupational therapists provide a wide range of interventions in various clinical settings aiming to enable individuals living with CP to perform their daily occupations. Support is required to enhance some aspects of their clinical process, such as using standardized evaluations and implementing interventions directly targeting healthy lifestyle adjustments. Building new evidence and advocating for the recognition of their role in CP management are essential to address the challenges of living with CP at each level of care.

## Key Messages

- Occupational therapists enable healthy lifestyle adjustments with persons living with CP. In Quebec, they are involved in each level of care, mostly in private practice and secondary care.
- Consistent with their role, occupational therapists use assessment and intervention methods to directly address the challenges of living with CP.
- Future action is required to rely on sound scientific evidence to articulate and justify occupation-based interventions.
